# Composition of the Frenkel–Kontorova and Ising models for investigation the magnetic properties of a ferromagnetic monolayer on a stretching substrate

**DOI:** 10.1038/s41598-021-00849-8

**Published:** 2021-11-02

**Authors:** Sergey V. Belim, Ilya V. Tikhomirov

**Affiliations:** Physics Department, Omsk State Technical University, Omsk, 644050 Russia

**Keywords:** Nanoscale devices, Magnetic devices

## Abstract

In the article, computer simulation on the behavior of a ferromagnetic thin film on a non-magnetic substrate by computer simulation is performed. The substrate is described by the two-dimensional Frenkel–Kontorova potential. The Ising model is used to describe the magnetic properties of a two-dimensional ferromagnetic film. The Wolf cluster algorithm is used to model the magnetic behavior of the film. A square lattice is considered for an unperturbed ferromagnetic film. Computer simulations show that mismatch of film and substrate periods results in film splitting into regions with different atomic structures. Magnetic properties for the obtained structure have been investigated. The hysteresis loop is calculated using the Metropolis algorithm. Deformations of the substrate lead to a decrease in the phase transition temperature. The Curie temperature decreases both when the substrate is compressed and when stretched. The change in phase transition temperature depends on the decreasing rate of exchange interaction with distance and the amplitude of interaction with the substrate. When the substrate is compressed, an increase in the amplitude of the interaction between the film and the substrate results in an increase in the phase transition temperature. The opposite effect occurs when the substrate is stretched. The hysteresis loop changes its shape and parameters when the substrate is deformed. Compression and stretching of the substrate results in a decrease in coercive force. The reduction in coercive force when compressing the substrate is greater than when stretching. The magnetization of the film is reduced by deformations at a fixed temperature.

## Introduction

Heterostructures consisting of a thin ferromagnetic film on a stretching substrate are actively used in spintronics devices. Interest in these structures is due to the magnetoelectric effect observed in them. The magnetoelectric effect allows the magnetization of the ferromagnetic film to be controlled by an electric field applied to the system. Control to the ferromagnetic film state is possible due to the interaction between the film atoms and the substrate. Changes the ferroelectric substrate crystal lattice in the electric field lead to deformations in the film crystal lattice. The magnetization and Curie point for the ferromagnetic change due to the magnetostriction phenomenon. The ability to control the ferromagnetics magnetization allows you to influence the amount of giant magnetic resistance in multilayer structures used in spin valves. The use of the magnetoelectric effect in spintronic devices significantly increases their energy efficiency.

The effect of the stretching substrate on the magnetic film has recently been actively investigated experimentally. In article^1^ ultrathin films of platinum on a ferroelectric substrate of ${\text {BaTiO}}_3$ are investigated. The article shows that the change in polarization and deformation of the substrate lead to a significant change in the film magnetization. The direct and reverse magnetoelectric effect for Ni films on ferroelectric substrates ${\text {BaTiO}}_3$ was investigated in operation^2^. Article^3^ examines the magnetoelectric effect in perpendicular magnetized ultra-thin films Pt/Co/Ta on a ferroelectric substrate. It has been shown that electric fields significantly affect the structure of magnetic domains. This indicates a giant magnetoelectric effect. Measurements by X-ray diffraction and X-ray reflectivity suggest the association of magnetoelectric effect with system deformations. In^4^, thin ferromagnetic films ${\text {CoFe}}_2{\text {O}}_4$/${\text {Pb}}({\text {Zr}}_{0.52}{\text {Ti}}_{0.48})$/${\text {LaNiO}}_3$ on a Pt/Ti/${\text {SiO}}_2$/Si substrate were experimentally investigated. For these systems, the ferromagnetic and ferroelectric phases coexistence at the interface of two media is observed. As a result, a large magnetoelectric effect is observed.

Theoretical studies of the magnetoelectric effect are focused on specific substances. In article^5^ on the basis of microscopic model and functions Green theory the substrate influence on thin films magnetic properties ${\text {BaCoF}}_4$ is studied. Magnetization and magnetic phase transition temperature decreases for compressible substrate ${\text {Al}}_2{\text {O}}_3$. For the tensile substrate MgO temperature and magnetization decrease. Article^6^ investigated the effect of order parameter fluctuations on two-dimensional films ${\text {RMnO}}_3$ (R = Tb, Lu and Y). The study is carried out as part of a modified Landau model. It has been shown that interaction with ferroelectric substrate plays an important role in the transition from paramagnetic to ferromagnetic phase. The temperature shift was investigated for the transition from the ferromagnetic phase to the antiferromagnetic phase in a small electric field in magnetoelectric heterostructures by combination of thermodynamic modeling and calculations from the first principles^7^. A temperature change on 5 K is achieved in a small electric field 0.1 MV/m. Calculations from the first principles^8^ show that for ultrathin films of FePd/MgO (001) the magnetoelectric effect decreases with film thickness. Simulation the phase field showed the possibility of controlling the vortex chirality in a triangular nanomagnet located on a ferroelectric substrate^9^. The change in the state of the nanomagnetic is caused by substrates deformation. Alignment with antiferromagnetic layer allows controlling exchange displacement at their boundary. Calculations based on the density functional method make it possible to detect the surface magnetoelectric effect in ferromagnetic films *Fe*(001), *Ni*(001) and *Co*(0001)^10^. The external electric field results in a marked change in surface magnetization and surface magnetocrystalline anisotropy. The behavior of a thin ferroelectric film on a ferromagnetic substrate is described in a nonlinear thermodynamic theory^11^. The deformation of the ferromagnet changes the polarization of the ferroelectric film. In this approach, substrate deformations are described using phenomenological theory. Magnetoelectric effect coefficients are calculated numerically for specific substances.

This paper studies the effects a ferroelectric substrate on the magnetic properties of a ferromagnetic film by computer simulation. This article focuses on combining the spin model for ferromagnetic and the Frenkel–Kontorova model to describe the interaction of film with a substrate. Both these models have long been successfully used for computer modeling of various phenomena in solids. The combination of models is used when describing phenomena that require taking into account several factors. Sharing two heterogeneous models can lead to new qualitative conclusions. Quantum calculations (calculations from the first principles) for such systems cannot describe all statistical phenomena. Therefore, spin models remain relevant. The purpose of this paper is to develop a baseline model and demonstrate the agreement of its findings with experimental data. Additional interactions (magnetic anisotropy, dipole–dipole, interface spin–orbit interaction, Dzyaloshinsky–Moriya interaction, etc.) can be easily introduced into our model as additive terms. We use a simple Ising model, since it allows us to obtain basic patterns. The transition to a Heisenberg model or XY model also does not entails much difficulty.

## Model

The ground state for the system must be calculated to describe the magnetic behavior of the two-dimensional ferromagnetic film on the substrate. Interaction with the substrate can change the mutual arrangement for the film atoms. The substrate has a periodic structure. We describe the effect of the substrate on the ferromagnetic film using a two-dimensional periodic potential. We use the two-dimensional Frenkel–Kontorova potential^12^.

1 $$\begin{aligned} U_{sub}=\frac{A}{2}\sum _n \left( 1-\cos \left( \frac{2\pi x_n}{b}\right) \cos \left( \frac{2\pi y_n}{b}\right) \right) . \end{aligned}$$

*b* is the period of the substrate. *A* is the substrate potential amplitude. $(x_n, y_n)$ are the coordinates of the atom with the number *n*.

We use harmonic approximation to interact between atoms. A square lattice with period $a_0$ is used as the initial position for the film atoms without a substrate. The potential interaction energy of the film atoms is recorded in a harmonic approximation.

2 $$\begin{aligned} U_{int}=\frac{g}{2}\sum _n \left( \left( x_{n+1}-x_n-a_0 \right) ^2+\left( y_{n+1}-y_n-a_0 \right) ^2 \right) . \end{aligned}$$

g is an elastic constant.

The total system energy consists of interaction between atoms and interaction with the substrate.

3 $$\begin{aligned} U=U_{int}+U_{sub}. \end{aligned}$$

To find the ground state, we calculate the minimum potential energy.

4 $$\begin{aligned} U\rightarrow min. \end{aligned}$$

Axes of initial atoms lattice are located along coordinate axes *OX* and *OY*. By means coordinate transformation, it is easy to show that the substrate potential forms a square grid oriented at an angle $\pi /4$ to the axes OX and OY.

The Monte Carlo method is used to find a potential energy minimum potential energy. The initial state corresponds to the position of the film atoms without a substrate. After that, the atoms are alternately shifted from the equilibrium position by a random vector. Offset vector length is not more than $0.1a_0$. If the new position of the atom is energetically advantageous, then it is accepted, if not energetically advantageous, then the atom returns to its original position. After that, the transition to the next atom occurs. In one round of the lattice, an attempt is made to shift all atoms. Successive lattice rounds are performed until the atoms take a stable position. The ground state corresponds to the stable position of all atoms.

Calculations show that when the film and substrate periods coincide, the atoms are placed in the substrate minima. The film is not deformed. After that, we simulate the stretching and compression of the substrate. This process occurs when the ferroelectric substrate is placed in an electric field, or when the system is heated. When heated, deformations occur due to different thermal expansion of the substrate and film. The change in the substrate period *b* occurs during deformations.

5 $$\begin{aligned} b=\varepsilon a_0. \end{aligned}$$

$\varepsilon$ is the strain factor.

**Figure 1:**
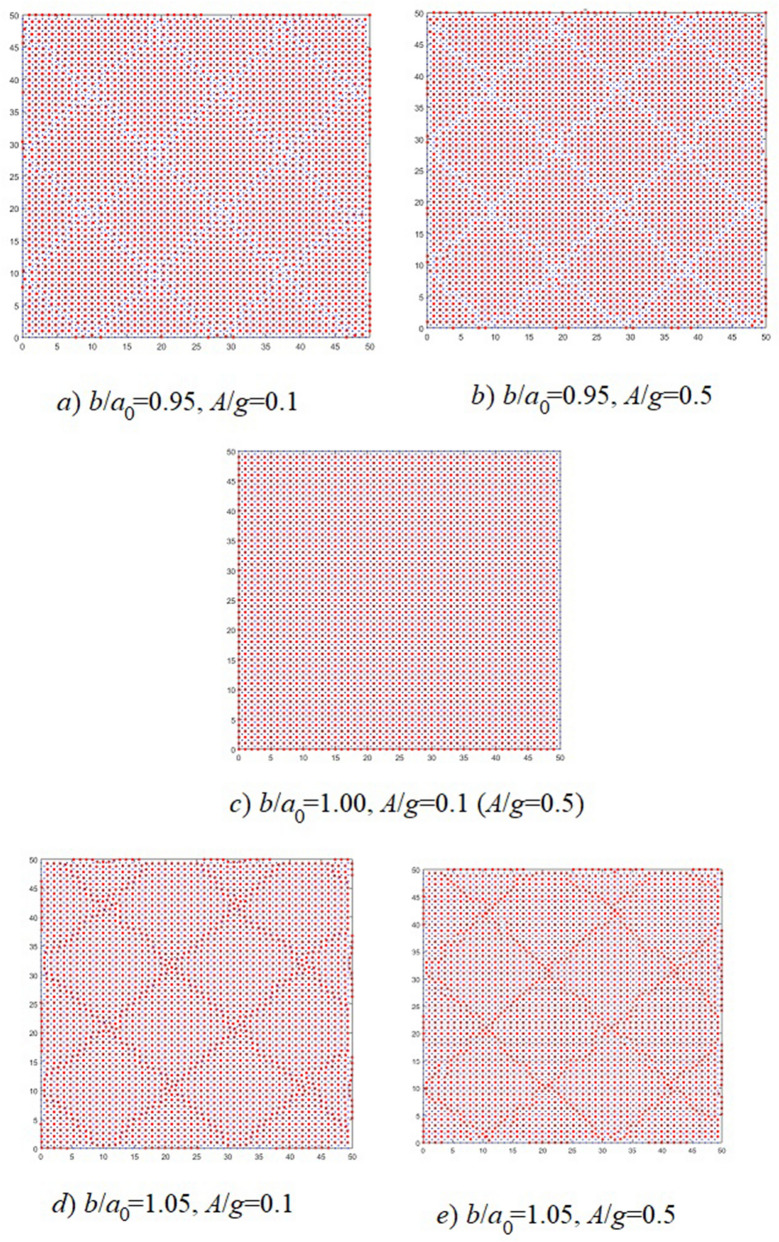
Placement of film atoms at different values substrate period *b* and potential amplitude *A* for a system with linear size $L = 50$.

We consider deformations no more than 5%. These strain values are observed in real experiments^1–4^. In our calculations, the deformation coefficient takes two values $\varepsilon = 0.95$ and $\varepsilon = 1.05$. Calculations are performed for substrate potential amplitude values $A=0.1$, $A=0.5$ and $A=1.0$. Three values are considered for the substrate period: $b=0.95a_0$, $b=a_0$, $b=1.05a_0$. Systems with linear dimensions $L = 50$ are simulated. The arrangement of film atoms for systems with size $L = 50$ and different values *A* and *b* are shown in Fig. 1.

As can be seen from Fig. 1, if the film lattices periods and the substrate are equal ($b = a_0$), then all the film atoms locates at the minima substrate potential. The film does not deform. If the film and substrate periods are different, non-uniform film deformations occur. The film is divided into regions, each of which has its own crystal lattice. Atoms are located in the minimum substrate potential energy inside the regions. The atoms arrangement between the regions depends on the ratio of the film and substrate periods. If the substrate period is less than the film period, compression within the region occurs. The distance between the formed regions increases. If the substrate period is longer than the film period, the resulting regions have a lower density than the original film. As a result, each region expands and their layering occurs. Between regions, the atoms density is greater than in the regions themselves. As a result, a film is formed on which a periodic superstructure is superimposed. The superstructure period depends on the ratio of film and substrate periods.

The Ising model is used to investigate the magnetic properties of a film on a substrate^13^. In this model, each atom has spin *S*, which can take one of two values ($+1/2$ or $-1/2$). Interaction occurs only between the nearest neighbors. The interaction between the spins is exchange. The exchange integral depends on the distance between atoms. The exchange integral decreases by exponential law.

6 $$\begin{aligned} J(|\vec {r}_i-\vec {r}_j|)=J_0\exp \left( -(|\vec {r}_i-\vec {r}_j|-a_0)/r_0\right) . \end{aligned}$$

$J_0$ is the exchange integral of an unperturbed lattice. The parameter $r_0$ shows the descending rate of the exchange integral with distance.

We consider only the short-range interaction with the exponential law in this model. Interaction between spins can be more complex in real systems^14–16^. The exchange interaction can be short-range and long-range. Long-range forces can lead to additional ordering and affect the phase transition temperature. Short-range forces may also depend on distance by non-exponential law. However, a model with an exponential law qualitatively correctly describes the behavior of the system in the first approximation. Other types of exchange interaction dependence on the distance between the spins lead to higher-order corrections to this model.

We write the Hamiltonian system in the external magnetic field.

7 $$\begin{aligned} H=-\sum _{(i,j)}J(|\vec {r}_i-\vec {r}_j|)S_iS_j+h\sum _i S_i. \end{aligned}$$

In the first term, summation is performed only on the nearest neighbors. *h* is the strength of the magnetic field. $r_i$ is the radius vector of an atom with the number *i*.

The state of the spin system in the Ising model can be described analytically for a two-dimensional square lattice^17^. There are solutions for some 3D systems^18,19^. For a spin-free system, an accurate analytical solution is not possible. Computer modeling is used for such systems.

The system magnetization is defined as the average spin value.

8 $$\begin{aligned} m=\left( \sum _i S_i\right) /N. \end{aligned}$$

N is the total number of spins in the system.

We use the Wolf cluster algorithm^20^ to computer simulate the phase transition in the system. Fourth order Binder cummulants^21^ are used to determine the phase transition temperature.

9 $$\begin{aligned} U=1-\frac{\langle m^4\rangle }{3\langle m^2\rangle ^2}. \end{aligned}$$

Triangular brackets are used to indicate the average value for thermodynamic states. Critical temperature is determined from the intersection point of fourth order Binder cummulants for systems with different linear dimensions^22^.

## Results

**Figure 2:**
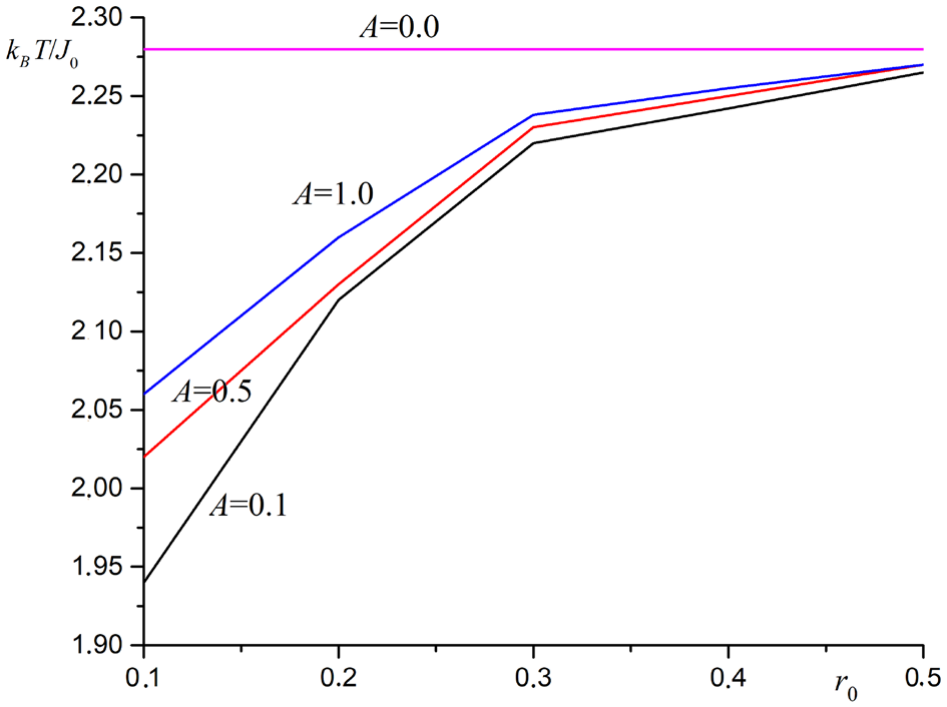
The graph for phase transition temperature versus parameter $r_0$ for substrate compression case ($b = 0.95a_0$) at different values of substrate potential amplitude.

The value $J_0 = 1$ is used in computer simulation. The parameter $r_0$ varies from $r_0 = 0.1$ to $r_0 = 0.5$ in increments of $\Delta r_0 = 0.1$. The graph of phase transition temperature versus parameter $r_0$ for substrate compression case ($b = 0.95a_0$) at different values substrate potential amplitude is shown in Fig. 2.

As shown in Fig. 2, the Curie temperature for the film is lowered when the substrate is compressed. At the same time, the Curie temperature increases with an increase in the parameter $r_0$. The decrease in the phase transition temperature is due to the division the film into regions. The interaction between the spins from different regions is much less than within the region. Also, this interaction is less than in the original film. The total exchange energy for the film spins is reduced. The increase in the phase transition temperature with an increase $r_0$ is explained by a slower decrease in the exchange integral with distance. When the distance between the spins changes, the decrease rate for the exchange integral is an important factor. The substrate potential amplitude significantly affects the Curie temperature at a rapid decrease in the exchange integral ($r_0=0.1$). The phase transition temperature increases as the substrate potential amplitude increases.

**Figure 3:**
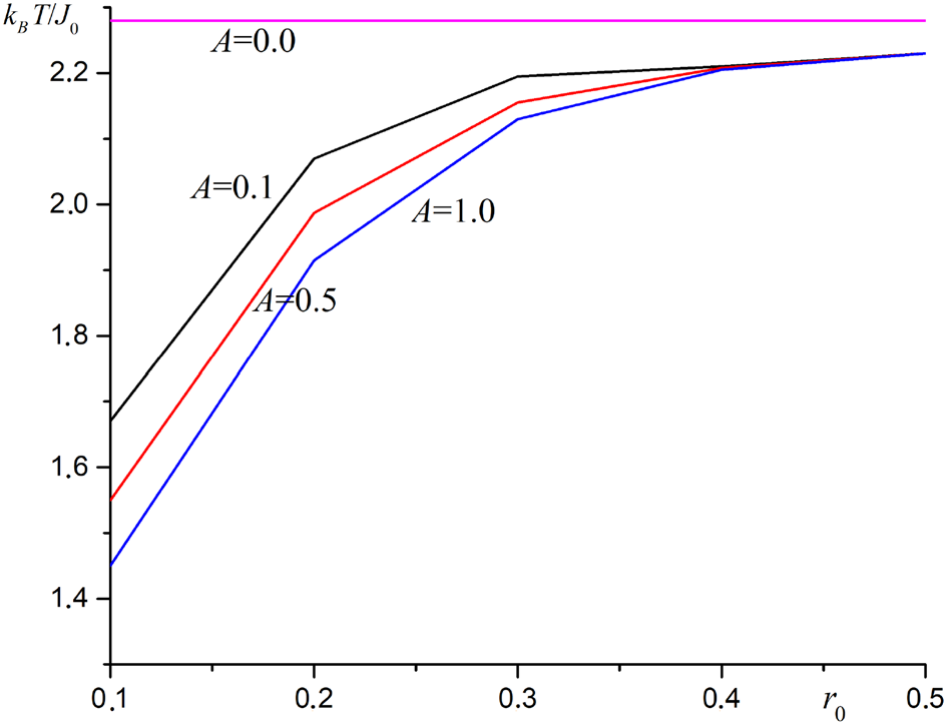
The graph for phase transition temperature versus parameter $r_0$ for the case substrate stretching ($b = 1.05a_0$) at different substrate potential amplitude values.

The graph for phase transition temperature versus parameter $r_0$ for the case substrate stretching ($b = 1.05a_0$) at different substrate potential amplitude values is shown in Fig. 3.

A decrease the phase transition temperature is observed when the substrate is stretched. This effect is also due to a decrease the total spin–spin exchange interaction energy. The phase transition temperature increases as the parameter $r_0$ increases. The dependence of the Curie temperature on the substrate potential amplitude is opposite to the compression case. As the substrate potential amplitude increases, the phase transition temperature decreases.

**Figure 4:**
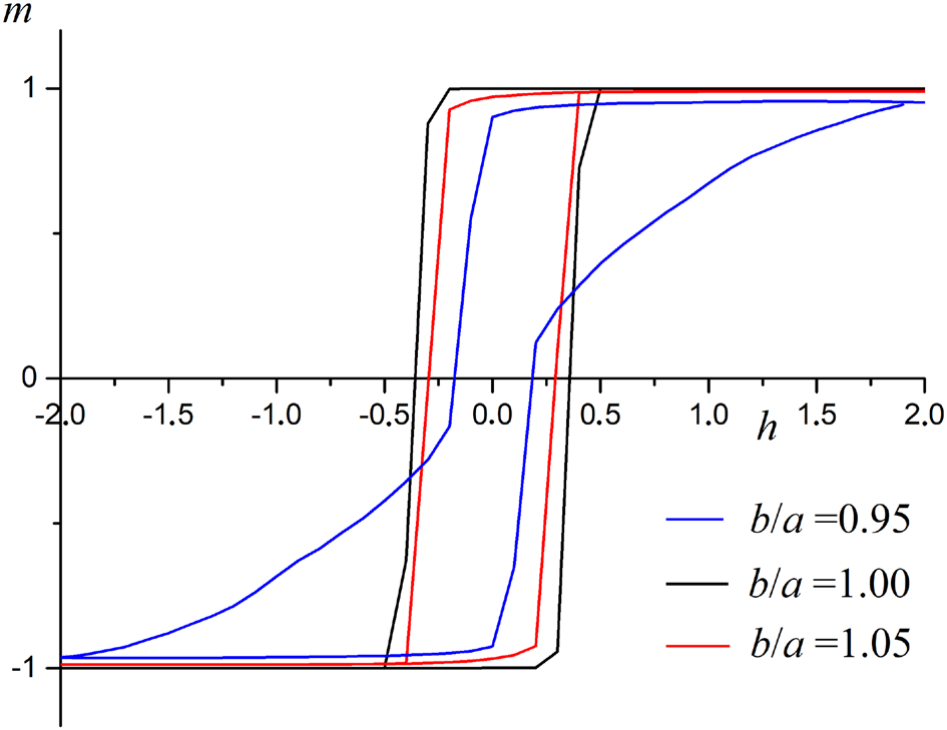
Hysteresis loops for three different film and substrate period ratios at $A=0.1$, $r_0=0.1$.

After that, we examined the behavior of the ferromagnetic film on the substrate in the magnetic field. We model a hysteresis loop at different system parameters. The Metropolis algorithm is used to calculate the hysteresis loop^22^. Wolf’s cluster algorithm destroys all information about the previous state and does not allow considering its effect on the current state. Hysteresis loop calculated for linear size system $L=50$. Figure 4 shows hysteresis loops for three different film and substrate period ratios.

As seen in Fig. 4, substrate deformations may result in a change in hysteresis loop shape and coercive force value. When compressed by 5%, coercive force decreases by 19% ($A=0.1$). When the system is compressed, the hysteresis loop area decreases. Magnetic reversal of a system requires smaller energy. This fact is due to the fact that the film breaks down into two-dimensional regions. These regions interact weakly with each other. When the substrate is stretched, the hysteresis loop shape changes. This change is due to the presence a periodic superstructure in which atoms concentration is increased. When stretching by 5%, the coercive force decreases by 49% ($A=0.1$).

**Figure 5:**
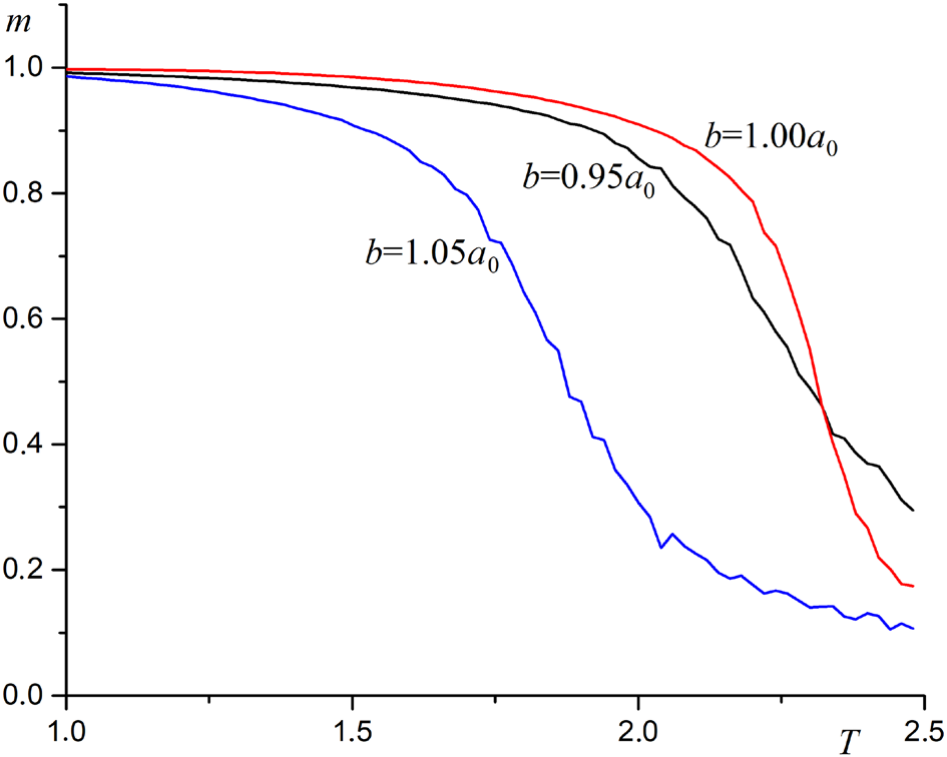
The dependence of the ferromagnetic film magnetization on temperature at various substrate deformation values at $A=0.1$, $r_0=0.1$.

Figure 5 shows the dependence of the ferromagnetic film magnetization on temperature at various substrate deformation values. At temperatures below the Curie point, substrate compression or stretching leads to a decrease the film magnetization. If the substrate is made of ferroelectric, this relationship corresponds to the observed magnetoelectric effect.

## Discussion

We investigated the effect of substrate deformations on the magnetic properties a two-dimensional ferromagnetic film bound to it. Strains at a fixed temperature approach or remove the system from the phase transition point. This bias results in a change the system magnetization. If the substrate is made of ferroelectric material, the deformations depend on the external electric field.

The effects obtained in the simulation correspond to experimental data. A decrease the phase transition temperature during both stretching and compression was observed for ferromagnetic film ${\text {La}}_{0.7}{\text {Ca}}_{0.3}{\text {MnO}}_3$ on ferroelectric substrate ${\text {BaTiO}}_3$^23^. Ni films on a single crystal substrate (011) -PMN-PT were investigated in Ref.^14^. It is shown that anisotropic deformations and change of crystal lattice lead to change of hysteresis loop for ferromagnetic film. In particular, a decrease the coercive force is observed. The greatest shift of the coercive force of 1253 Oe is achieved in the electric field of 10 kV/cm. Magnetization switching in film ${\text {Ni}}_{80}{\text {Co}}_{20}$ deposited on substrate Pb (Mg, Nb) O-3-PbTiO$_3$ under action of external electric field is investigated in article^15^. Substrate lattice deformations and magnetic moment were examined as a response to an external electric field. This system supports two magnetization states when the electric field is removed. Magnetoelectric interaction in heterostructure ${\text {Pb}} ({\text {Zr}}_{0.52}{\text {Ti}}_{0.48})/{\text {LaNiO}}_3/{\text {Ni}}$ studied in Ref.^16^. ${\text {LaNiO}}_3$ is a buffer layer for a 15 nm thick ferroelectric film. This system shows a large magnetoelectric voltage coefficient 560 ${\text {mV}}\; {\text {cm}}^{-1} {\text {Oe}}^{-1}$ at a relatively low magnetic bias field of 65 Oe.

Article^24^ examined the effect an electric field on the magnetic response of thin films ${\text {Fe}}_{89}{\text {Ga}}_{11}$ ($t = 6$, 11, 17, 22 and 28 nm) deposited on ferroelectric PMN-PT single crystals. When applying an electric field, hysteresis loops are modified, which is consistent with a positive magnetostriction constant. In article^25^ thin films ${\text {Ba}}_{0.9}{\text {Ca}}_{0.1}{\text {TiO}}_3/{\text {CoFe}}_2{\text {O}}_4$ on ${\text {Pt}}/{\text {Ti}}/{\text {SiO}}_2/{\text {Si}}$ substrates are studied. These structures have great magnetoelectric effect coefficient value (82 ${\text {mV}} \; {\text {cm}}^{-1} {\text {Oe}}^{-1}$).
